# Sensory and autonomic function and structure in footpads of a diabetic mouse model

**DOI:** 10.1038/srep41401

**Published:** 2017-01-27

**Authors:** Ying Liu, Blessan Sebastian, Ben Liu, Yiyue Zhang, John A. Fissel, Baohan Pan, Michael Polydefkis, Mohamed H. Farah

**Affiliations:** 1 Department of Neurology, Johns Hopkins University School of Medicine, Baltimore, MD, USA.

## Abstract

Sensory and autonomic neuropathy affects the majority of type II diabetic patients. Clinically, autonomic evaluation often focuses on sudomotor function yet this is rarely assessed in animal models. We undertook morphological and functional studies to assess large myelinated and small unmyelinated axons in the db/db type II diabetes mouse model. We observed that autonomic innervation of sweat glands in the footpads was significantly reduced in db/db mice compared to control db/+ mice and this deficit was greater compared to reductions in intraepidermal sensory innervation of adjacent epidermis. Additionally, db/db mice formed significantly fewer sweat droplets compared to controls as early as 6 weeks of age, a time when no statistical differences were observed electrophysiologically between db/db and db/+ mice studies of large myelinated sensory and motor nerves. The rate of sweat droplet formation was significantly slower and the sweat droplet size larger and more variable in db/db mice compared to controls. Whereas pilocarpine and glycopyrrolate increased and decreased sweating, respectively, in 6 month-old controls, db/db mice did not respond to pharmacologic manipulations. Our findings indicate autonomic neuropathy is an early and prominent deficit in the db/db model and have implications for the development of therapies for peripheral diabetic neuropathy.

Autonomic dysfunction is common in diabetes and affects many important functions including exercise tolerance, gut peristalsis, sexual function and cardiovascular health; yet it is often underappreciated. Cardiac autonomic dysfunction contributes to increased rates of sudden death in diabetes and prediabetes^1^, while reduced pedal sweating is integral to diabetic ulcer formation and poor wound healing^2^. The annual cost of diabetic neuropathy in the US was estimated at $10.9 billion (2001 dollars)^3^. In men, erectile dysfunction, in part caused by autonomic neuropathy, is often a presenting symptom of diabetes and heralds occult vascular disease. Methods to assess autonomic function are technically complex and results can be confounded by medications for common disorders (hypertension)^4^, diet (coffee) and circadian patterns^5^. Cardiac autonomic assessment includes measurement of heart rate variability (HRV), blood pressure testing, and Valsalva maneuvers that require dynamic patient participation and cooperation^6^,^7^. These variables can complicate routine clinical autonomic assessment.

Animal models are an attractive tool to study autonomic function as they allow the opportunity to control many of the factors that complicate human autonomic measurements, yet, there have been relatively few such studies and most have focused on heart rate control. In contrast to human diabetic cardiac autonomic neuropathy in which patients typically exhibit tachycardia^8^, most experimental studies have demonstrated bradycardia^9^,^10^,^11^,^12^. In type 1 (streptozotocin-induced) diabetic rodents, abnormalities in autonomic axons have been reported, including dystrophic noradregenergic axons in mesenteric nerve^13^,^14^,^15^.

Dependent on the model, genetic background, and the time of assay, mouse models of diabetic sensory neuropathy are known to develop both hyper- and hypo-sensitivity to thermal stimuli, loss of sensory fibers in the footpads, and reduced nerve conduction^16^,^17^,^18^,^19^,^20^,^21^. Type I diabetes is most often modeled by streptozotocin-mediated β-cell ablation in rodents, resulting in reduced epidermal innervation and sensory neuropathy^22^,^23^,^24^. Rodent models of type II diabetes include, among others, the widely used models leptin-deficient ob/ob mice and leptin receptor-deficient db/db mice^25^,^26^. The db/db mice exhibit features of neuropathy, such as decreased nerve conduction velocity^27^, axonal atrophy^28^, and reduced epidermal innervation^20^. Studies on these models have contributed to our understanding of pathogenesis of diabetic sensory neuropathy^21^,^29^. Whether db/db mice exhibit autonomic neuropathy has not been explored.

Here, we describe a combined approach in which pathological assessment of footpad autonomic innervation is measured and correlated with functional assessment of footpad sweating. We observed that small unmyelinated autonomic fibers were preferentially affected early in disease compared to large myelinated fibers and that autonomic fibers innervating sweat glands were more prominently affected than their unmyelinated epidermal sensory counterparts at early time points. Pharmacologic manipulation of sweat production was possible with pilocarpine and glycopyrrolate in db/+ animals, however db/db animals were unresponsive to such treatments at 6 months of age.

## Results

We first examined the db/db mice in our colony for body weight and blood glucose level in order to establish known features of diabetes. Beginning at the age of 4 weeks, db/db mice had significantly higher body weight than db/+ mice and continued to gain weight steadily until 12 weeks of age, after which body weight plateaued (Supplementary Fig. 1). The blood glucose levels were significantly elevated in db/db mice from 4 weeks until 20 weeks of age, at the last recorded time point (Supplementary Fig. 1). Thus, our colony of db/db mice exhibited well-established features of diabetes.

**Figure 1:**
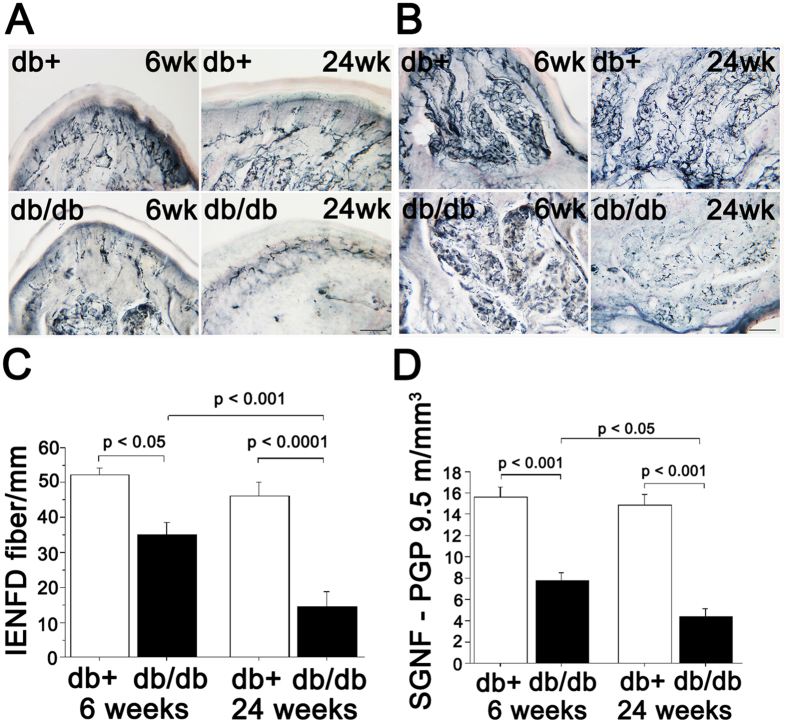
Reduction in sweat gland innervation is more severe than loss of epidermal fibers in footpads of db/db mice at early stage. (**A**) Representative images showing the innervation of epidermis from the footpads of db/db (bottom) and db/+ (top) mice at 6 weeks (left) and 24 weeks (right) of age. Nerve fibers were visualized by staining for pan-neuronal marker PGP 9.5. Innervation of footpads of db/db mice is reduced compared with age-matched control db/+ footpads. (**B**) Representative images showing innervation of sweat glands from the footpads of db/db (bottom) and db/+ (top) mice at 6 weeks (left) and 24 weeks (right) of age. Nerve fibers were visualized by staining for pan-neuronal marker PGP 9.5. Innervation of sweat glands of db/db mice is reduced compared with age-matched db/+ sweat glands. For A and B, scale bar = 100 μm. (**C**) Quantification of PGP 9.5-postive fibers of intraepidermal nerve fiber density (IENFD) in db/+ and db/db mice (n = 6 per age group). IENFD is significantly decreased in db/db mice compared to db/+ mice (33% in 6 weeks old group, 68.9% in 6 months old group). (**D**) Quantification of PGP 9.5-postive fibers of sweat gland nerve fiber density (SGNFD) in db/+ and db/db mice (n = 6 per age group). SGNFD is significantly decreased in db/db mice compared to db/+ mice (50.7% in 6 weeks old group, 70.4% in 6 months old group).

**Figure 2:**
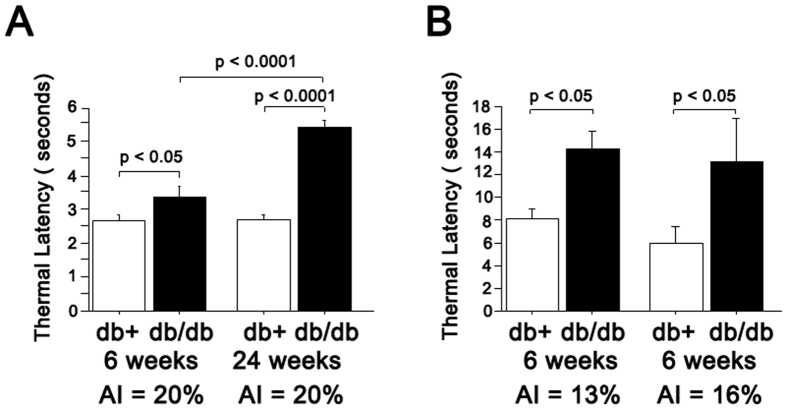
Paw withdrawal latency of mice to a thermal stimulus on hindpaws. (**A**) At intensity of 20%, the thermal latency was significantly delayed in db/db mice at 6 weeks (n = 14, 3.36 ± 0.31 sec) and 24 weeks (n = 11, 5.43 ± 0.22 sec) compared to db/+ mice at 6 weeks (n = 8, 2.66 ± 0.178 sec) and at 24 weeks (n = 8, 2.7 ± 0.22 sec). p < 0.05 in 6 weeks group and p < 0.0001 in 24 weeks group. (**B**) At lower intensities (13% and 16%), the thermal latency was significantly delayed in db/db mice at 6 weeks compared to age-matched db/+ mice.

**Figure 3:**
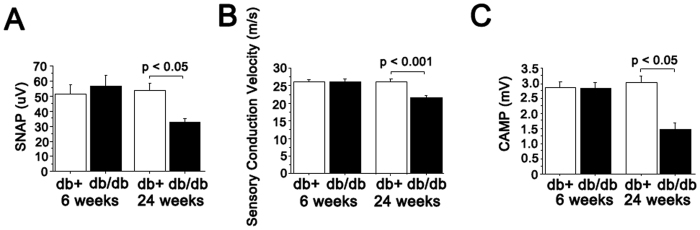
Nerve physiology investigations of db/+ and db/db at 6 and 24 weeks of age. (**A**) Amplitudes of sensory nerve action potential (SNAP) in tail nerves are not statistically different between db/+ and db/db mice at 6 weeks of age, but at 24 weeks age, SNAP of db/db mice is significantly reduced compared with that of db/+ mice. (**B**) Sensory nerve conduction velocity in 24 week old db/db mice was significantly decreased (21.6 ± 0.36 m/s) compared to same age db/+ mice, but there is no significant deficit in 6 week old db/db mice. (**C**) Amplitudes of compound muscle action potential (CMAP) is significantly reduced in 24 week-old db/db mice, but not in 6 week-old db/db mice compared with age matched db/+ mice. For 6 weeks, n = 8 db/+; n = 14 db/db and for 24 weeks, n = 8 db/+; and n = 11 db/db mice.

The db/db mice have been shown to have features of diabetic sensory neuropathy at 24 weeks of age^20^. We examined the pathology (intraepidermal nerve fiber density (IENFD)) and function (sensory nerve conduction velocity and latency of hind paw response to heat stimuli) in db/db mice starting at 6 weeks of age to establish the earliest age at which these mice show neuropathy. The IENFD in db/db animals was significantly reduced by 33% compared to control db/+ mice at 6 weeks, and further decreased to 68.9% loss at 24 weeks (Fig. 1A,C and Supplementary Table 1). At 6 weeks of age, db/db mice showed a delayed withdrawal response to radiant heat applied to the footpads at high (20%) intensity (Fig. 2A). To exclude the possibility that the delay response was a result of db/db mice being prone to tissue damage from high intensity heat beam, we tested the 6 week old mice at lower intensities (13% and 16% beam intensities). The db/db mice had significantly delayed responses (Fig. 2B) at lower intensities, indicating that delayed responses to noxious stimuli correlate with partial loss of innervation as early as 6 weeks of age (Fig. 1A–C). At 6 weeks, there were no statistically significant differences in sensory nerve action potential (SNAP) amplitudes or tail nerve conduction velocities between db/+ and db/db mice (Fig. 3A–C). In contrast, at 24 weeks, db/db mice exhibited significantly reduced sensory conduction velocity and SNAP compared to db/+ mice (Fig. 3A–C), a finding consistent with previously published work^20^. Similarly, compound muscle action potentials (CMAP) did not significantly differ between db/+ and db/db mice at 6 weeks, but were significantly reduced in db/db mice at 24 weeks (Fig. 3C). Together, these data indicate that significant sensory (PGP-positive epidermal fibers) denervation in footpads correlates with reduced sensitivity to heat stimuli at the same site (footpads), but deficits in sensory nerve conduction and action potential amplitude manifest at a later stage of neuropathy in db/db mice.

To characterize structural changes in autonomic nerve fibers, we examined the sweat glands in the footpads for degree of innervation. The density of PGP-positive fibers in the sweat glands in db/db mice was significantly reduced to 50.7% of control db/+ mice levels at 6 weeks (Fig. 1B,D and Supplementary Table 1). By 24 weeks, fiber density was decreased by 70.4% in sweat glands of db/db mice (Fig. 1B,D and Supplementary Table 1). Additionally, we observed a qualitatively dramatic and quantitatively significant reduction in tyrosine hydroxylase (TH) staining in the sweat glands of db/db mice (Supplementary Fig. 2A,B). We conclude that there is a more prominent reduction in sweat gland innervation compared to loss of sensory fibers in db/db mice (50.7% sweat gland innervation vs 33% sensory innervation), particularly at early stages (Supplementary Table 1).

**Figure 4:**
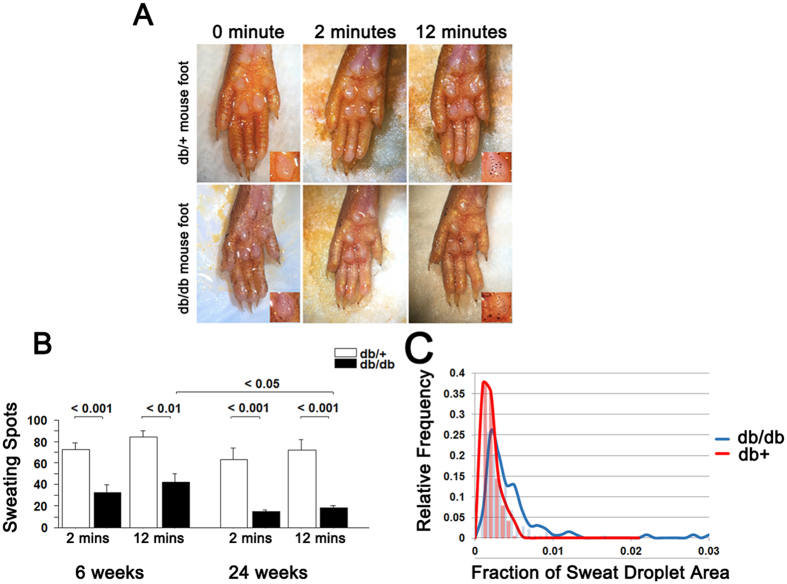
Deficits in sweating in the footpads of db/db mice. (**A**) Representative images of sweat droplets (dark precipitates from iodine/starch assay) on footpads of db/+ and db/db mice. The db/db footpads have fewer droplets that are larger and more variable in size. Insets show a single sweat gland. (**B**) Quantification of sweat droplets at 6 and 24 weeks of age in db/+ and db/db mice. In db/db footpads, sweat droplet number is significantly decreased at 6 weeks of age compared to control db/+. While the numbers of droplets do not change with age in db/+, sweat droplet number does decrease significantly with diabetes duration (from 6 to 24 week of age) in db/db mice. (**C**) The db/db mice have sweat droplets that are more variable in size and whose size distribution is significantly skewed to the right. The fraction of sweat drop area represents size of individual spots divided by pad area for pads 3 and 4 (n = 10 per group for 6 weeks, n = 8 for db/db at 24 week, and n = 10 for db/+ at 24 weeks of age).

We next examined whether the reduction in innervation of sweat glands led to physiological abnormality in sweat production. We utilized an iodine/starch based sweat assay^30^. In this assay, footpad perspiration appears as dark precipitates on iodine and starch coated footpads. Digital images were obtained, blinded, and subsequently analyzed for sweat droplet number and size, analogous to strategies used in human studies^31^ and demonstrated to yield equivalent results as silicone imprints^31^. Formation of dark spots in footpads of db/+ and db/db mice was monitored every two minutes for 12 minutes. At 6 and 24 weeks, db/db mice had significantly fewer sweat droplets (dark spots) compared to db/+ mice (Fig. 4), indicating a deficit in sweat production likely due to reduced autonomic innervation (Fig. 1B and D). Sweat droplet number correlated with sweat gland nerve fiber density (SGNFD) in both groups of animals at 6 and 24 weeks, p < 0.05. Furthermore, the rate of sweat droplet formation was slower in db/db mice compared to db/+ mice at 6 months (0.17 + 0.12 vs. 0.34 + 0.12 droplets/min, p < 0.05). Additionally, db/db mice had larger sweat droplets compared to db/+ animals (0.0052 + 0.0051 vs 0.0015 + 0.0010 mm^3^, p < 0.0001) and size of the sweat droplets had a normal distribution in the db/+ mice, while the db/db animals had many large sweat droplets with the distribution curve being shifted to the right (Fig. 4C).

**Figure 5:**
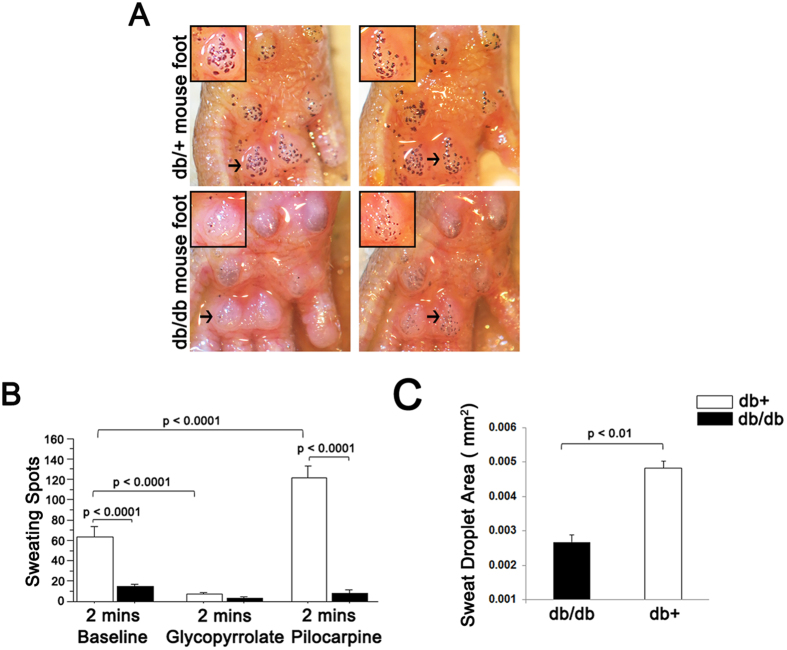
Deficits in foot pad sweating after pharmacologic stimulation. (**A**) Representative images of sweat droplets (dark precipitates from iodine/starch assay) on footpads of db/+ and db/db mice after administration of pilocarpine. The db/db footpads have fewer and smaller droplets. (**B**) Sweat function in db/db mice is less responsive to pharmacologic manipulations than in control db/+ animals. The number of sweat droplets changes significantly in response to administration of glycopyrrolate (significant reduction) and pilocarpine (significant increase) in db/+ but not in db/db mice. (**C**) Sweat droplets in db/db mice were significantly smaller after pilocarpine administration compared to db/+ controls (n = 10 db/+ and n = 8 db/db.

We tested the responsiveness of sweat gland function in db/db mice to pharmacologic manipulation at 6 month of age. Unlike control db/+ mice that readily responded to glycopyrrolate (sweating inhibitor) and pilocarpine (enhancer of sweating) (Fig. 5A,B), sweating in db/db mice was not significantly affected by these compounds after 2 minutes (Fig. 5B) or 12 minutes (data not shown), a finding consistent with sweating dysfunction in db/db mice being linked to a reduction in autonomic innervation. Sweat droplets after pilocarpine stimulation were also significantly smaller in db/db animals compared to db/+ controls (Fig. 5C) as has been reported in human studies^31^,^32^.

## Discussion

Diabetic peripheral neuropathy is the most common complication of diabetes, affecting 60–70% of diabetes patients (http://diabetes.niddk.nih.gov/dm/pubs/neuropathies). The salient features are sensory and autonomic dysfunction. Rodent models of diabetes have facilitated current understanding of the pathobiology of diabetic sensory neuropathy^16^,^17^,^20^,^21^,^29^. Autonomic dysfunction, particularly sudomotor function, has not been as extensively studied in rodent models^33^. Here, we report that autonomic innervation of sweat glands is greatly compromised, both morphologically and functionally, in db/db mice. These studies reveal that db/db mice could serve as a model to rigorously investigate pathogenesis of autonomic neuropathy as well as a complementary outcome measure to assess potential treatments in the future.

Previous studies on diabetic autonomic neuropathy in rodent models have mainly examined cardiac abnormalities, though in general, few studies have investigated sudomotor dysfunction relative to other complications of diabetes^33^. Changes in the enteric nervous system have been elegantly described in the type I STZ diabetic model, though this contrasts with human disease where the majority of diabetes is type II and clinical autonomic assessments typically concentrate on blood pressure and sudomotor function^13^,^14^. In db/db mice, cardiac innervation by sympathetic fibers is reduced at 6 month of age^34^, and cardiac autonomic function is abnormal beginning at 8 weeks of age^35^,^36^,^37^. Definitive conclusions about pathogenesis of diabetic autonomic neuropathy of the heart, however, are complicated by the presence of diabetic cardiomyopathy in which the primary effects of diabetes are on cardiomyocytes themselves^38^,^39^,^40^,^41^. In the present study, we sought to systematically assess sweat gland innervation and function in order to investigate temporal development of diabetic autonomic neuropathy in mouse models.

The development of reproducible skin biopsy protocols has aided accuracy for assessing diabetic neuropathy progression in human patients^42^,^43^,^44^,^45^,^46^,^47^,^48^,^49^,^50^. Building upon observations by Gibbons and Kennedy^51^,^52^,^53^, we assessed by stereology, sweat gland innervation density in human skin biopsies and found that sweat gland innervation was severely reduced in subjects with diabetes compared with controls and was inversely correlated with neuropathy impairment score of the lower limb (NIS-LL)^51^,^52^,^53^,^54^,^55^,^56^. Those results motivated us to investigate sweat gland innervation and function in db/db mice to develop a reliable and reproducible animal system for diabetic autonomic neuropathy. Sweat glands in footpads can be easily identified in mice as they lay beneath the dermis. Thick (50 micron) sections perpendicular to the footpad surface are sufficient to systematically quantify both sensory (IENFD) and autonomic (underlying sweat gland) innervation (Fig. 1). This enables the comparison of the loss of different fiber types in close proximity and thereby exclude confounding factors such as regional viability in innervation for fiber types.

Both sweat gland innervation (Fig. 1) and sweating (Fig. 4) were deficient in db/db mice as early as 6 weeks of age, suggesting that autonomic neuropathy manifests very early in db/db mice. This is consistent with human studies that reported autonomic alterations in patients with prediabetes^57^. Though not as prominent as autonomic fiber loss, a reduction in unmyelinated sensory fibers (IENFD) was also evident at 6 weeks of age in db/db (Fig. 1). Electrophysiologically, nerve conduction studies were not altered at this age, but latency to thermal sensation was delayed at 6 weeks of age (Fig. 2). This might indicate that fiber loss is restricted to terminal nerve endings at sites of innervation. Together our finding of deficits in autonomic innervation and function in db/db mice provides novel tools to study the pathogenesis of and potential interventions for diabetic autonomic neuropathy.

Sweat droplet number and sweat gland innervation were both reduced at 6 weeks and 6 months in db/db animals providing agreement between measures of structure and function. Only humans and nonhuman primates sweat to control body temperature and the sweat droplet formation that we measured was not stimulated by a rise in body temperature as with thermoregulatory sweat testing (TST) in humans. Such testing provides a combined measure of pre and postganglionic sudomotor function and when combined with a postganglionic test can localize the site of dysfunction^55^. In the present study, sweat gland droplet formation represents a postganglionic measurement. Therefore, these results do not provide insight into preganglionic dysfunction. Consistent with this, the severity of reduction as measured by either measure (SGNFD and sweat droplet formation) were nearly identical at both time points. The rate of sweat droplet formation was reduced in db/db mice at 6 months compared to db/+ animals and is consistent with dynamic measurements of sweat function performed in humans^31^. Without pharmacologic provocation, sweat droplet size was increased in db/db mice, perhaps consistent with autonomic dysfunction. The response to pilocarpine stimulation was muted in db/db animals with fewer and smaller sweat droplets compared to non-diabetic animals. This pattern is consistent with human studies, which similarly measured sweat production after pharmacologic stimulation^31^,^32^,^58^. These consistencies across species supports the validity of the model.

We also assessed the effect of pilocarpine and glycopyrrolate on sweat production. As expected, nondiabetic db/+ animals demonstrated increased and decreased sweat production respectively, in response to these two agents. In contrast, there was little response to either agent in the 6 month old db/db animals. These results suggest that sudomotor function in long duration diabetes is severely impaired and is consistent with epidemiological studies demonstrating reduced HRV in diabetes in cross sectional studies^59^,^60^,^61^,^62^,^63^ as well as a progressive decline in autonomic function among those with diabetes versus those without diabetes^64^.

There are several notable limitations to this study. We only studied a single model of type II diabetes and only included male animals. Mice do not regulate body temperature through sweating, thus we were unable to assess preganglionic sudomotor function.

In conclusion, the results in this paper support a spectrum of nerve fiber involvement in diabetes with unmyelinated small caliber nerve fibers being affected before large myelinated fibers. Among unmyelinated fibers, postganglionic sudomotor fibers were affected more prominently than sensory fibers (IENFD). Sweat gland innervation correlated with sweat droplet number and the latter was responsive to pharmacologic manipulation in control but not diabetic animals. Sudomotor function and sweat gland innervation declined with increasing diabetes duration. Together these results provide a tool with which to study the autonomic nervous system in experimental diabetes and assess promising therapies.

## Material and Methods

### Animal

Male db/+ and db/db (BKS.Cg-DOCK7 background) mice were purchased from Jackson Labs (Bar Harbor, Maine, USA). A total of 36 db/+ and 45 db/db mice were used in this study. All experiments and animal care procedures were conducted in accordance with the guidelines of the Johns Hopkins University Committee on the Use and Care of Animals. All mice associated with this study were housed in a facility at Johns Hopkins University and all experimental protocols were approved by Johns Hopkins University’s animal care and use committee.

### Electrophysiological studies

Nerve electrophysiology studies were performed with the aid of Power Lab signal acquisition setup (AD Instruments, Colorado Springs, CO, USA). Mice were anaesthetized using 2.5% isoflurane using a nose cone (Isosol, Vedco, St. Joseph, MO). Body temperature was maintained at 34–38 °C by placing the mice on a warmed blanket. Temperature was monitored throughout the testing procedure and confirmed to be >34 °C prior to obtaining any measurements. Orthodromic tail sensory nerve conduction velocity (distance between stimulating and recording anode electrodes/latency) was measured using the smallest current that resulted in a maximal amplitude response with a 0.1 ms duration. The stimulating and recording electrode anodes were separated by a standard distance (50 mm) with the recording electrode at the tail base. A ground was placed between the stimulating and recording electrodes. Amplitudes of the evoked potentials were recorded. The “peak-to-peak” latency of the potentials was recorded with the average of 16 stimuli used as the final value. CMAPs were measured by stimulation with subdermal needle electrodes placed near the sciatic nerve at the sciatic notch and surface recording electrodes over tibial nerve-innervated intrinsic foot muscles. Recordings were made with supramaximal stimulation (~10 V). The the latencies, negative peak amplitudes, and durations of the sciatic CMAPs were recorded.

### Latency of thermal sensation

Latency to noxious thermal stimuli was measured in hindpaw using radiant heat, Hargraeves method (IITC Life Sciences Model 400 heated base, Woodlands Hills, CA). Mice kept in plexiglass restrainers were pre-acclimated for 40 minutes on (32 °C) clear platform prior to testing. A beam of light at defined (13%, 16%, or 20%) intensities were shined on the hindpaw of mice on preheated (32 °C) clear platform. The time it took each mouse to withdraw hindpaw was scored as latency to noxious thermal stimulus. For each mouse, 6 trials, at 20 minute intervals, were scored to obtain an average response.

### Sweat assay

Mice were anesthetized using 2–3% isoflurane (Isosol, Vedco, St. Joseph, MO). Left hind paws were cleaned and painted with 3.5% iodine (Sigma-Aldrich) in ethanol, allowed to dry, and followed by coating with 10% starch solution in costar oil (both from Sigma-Aldrich). Footpad photographs were taken at a standard distance and magnification with an 8 megapixel digital camera. Images were obtained at 2, 4, 8, and 12 minutes to document the dark precipitates formed by sweat droplet formation. Each image contained a 1 mm^2^ reticle to determine droplet size. The number of dark spots were counted for each paw. The rate of sweat formation was determined by the slope of the regression of time (minutes) vs. sweat droplet number.

Responsiveness to pharmacologic stimulation in 6 month old mice was assessed with glycopyrrolate and pilocarpine. Glycopyrrolate (0.25 mg/kg) was given subcutaneously and sweat function was assessed 30 minutes later. 10 db/+ mice and 5 db/db mice were studied. Pilocarpine (50 ug, in 5 uL 0.9% saline) was injected subcutaneously into the footpad and sweat function was measured 2 minutes later. Droplet size was determined from footpad images using Stereo Investigator. Individual droplets were manually traced for footpads 3 and 4 and the area of each droplet automatically calculated from a reticle reference. Droplet size and distribution were compared from db/db and db/+ animals after adjusting for footpad size.

### Immunohistochemistry

Mice were euthanized with CO_2_ and hind limbs at the ankle were collected and were fixed in Zamboni fixative (Newcomers Supply, Middleton, WI) for 24–72 hours. Footpads were dissected out and were washed in phosphate buffer and placed in cryoprotectant (30% glycerol) solution. Tissue blocks were cut by freezing microtome at 50 μm intervals and immunohistochemical staining was performed using a standard chromogen technique with the following antibodies: rabbit anti-PGP 9.5 (AbD Serotec, a Bio-Rad Company, Kidlington, UK) and rabbit anti-TH (Novus Biologicals, Littleton, CO). For each marker, four 50 micron sections were selected from footpads 3 and 4. These were selected at random from throughout the possible sections. Sections were incubated over night at room temperature in 96 well tissue culture plates on a horizontal tabletop shaker at 50 rolls per minute. The following day, sections were washed in phosphate buffer 2–3 times and then incubated with biotinylated goat anti-rabbit Ab (Vector Labs, Burlingame, CA) for 2–3 hr. Bound immunoglobulin was visualized by the ABC kit (Vector labs, Burlinggame, CA).

### IENFD analysis

Individual PGP 9.5 positive intra-epidermal nerve fibers crossing the dermal-epidermal junction were counted. IENFD was calculated by dividing the number of counted fibers by the length of epidermis and expressed as fibers/mm.

### Stereological analysis of axon density in sweat glands

The density of PGP 9.5 positive axons in sweat glands of footpads were measured by stereological length estimation using spherical probes option (based on an unbiased fractionator sampling methodology), Stereo Investigator (MicroBrightField, Williston, VT). Using a high-power objective lens with a high numerical aperture (X100/1.25), the top and bottom of the footpad sections were identified, and the thickness of the mounted sections was determined. A virtual hemisphere with a dissection height of 15 μm and a guard zone of 1 μm on both surfaces was used. The largest portion of the gland contour was traced using X10/0.30 Plan Neofluar objective of a Zeiss light microscope (Carl Zeiss Meditec, Dublin, CA). A program-generated grid ensured systematic random sampling of sites. Under X100/1.25 at each site, a virtual hemisphere of constant volume was delivered through the z-axis plane with the stage controlled by the program. The structure was counted as a “profile” when a fiber transected the hemisphere boundary. Sweat gland volume was calculated using the Cavalieri method that we previously used for human skin biopsies^54^. All measurements were obtained using DAT files, and sweat gland innervation was expressed as m/mm^3^ for each sweat gland.

### Statistical analysis

Comparisons between multiple groups were made by two-way ANOVA. Pairwise comparisons were made using Student’s t-test. Prism 6.0 (GraphPad, La Jolla, CA) was used to perform the analyses and any value of p < 0.05 was scored as statistically significant. Data are presented as mean ± SEM. The frequency distribution of sweat droplet size was plotted using Stata 11.0 (College Station, TX) and smoothed curves were generated by Excel.

## Additional Information

**How to cite this article:** Liu, Y. *et al*. Sensory and autonomic function and structure in footpads of a diabetic mouse model. *Sci. Rep.* **7**, 41401; doi: 10.1038/srep41401 (2017).

**Publisher's note:** Springer Nature remains neutral with regard to jurisdictional claims in published maps and institutional affiliations.

## Supplementary Material

Supplementary Dataset 1

## References

[b1] Laukkanen J. A., Makikallio T. H., Ronkainen K., Karppi J. & Kurl S. Impaired fasting plasma glucose and type 2 diabetes are related to the risk of out-of-hospital sudden cardiac death and all-cause mortality. Diabetes Care 36, 1166–1171, doi: 10.2337/dc12-0110 (2013). 23248190 PMC3631879

[b2] Brem H., Sheehan P. & Boulton A. J. Protocol for treatment of diabetic foot ulcers. Am J Surg 187, 1S–10S, doi: 10.1016/S0002-9610(03)00299-X (2004). 15147985

[b3] Gordois A., Scuffham P., Shearer A., Oglesby A. & Tobian J. A. The health care costs of diabetic peripheral neuropathy in the US. Diabetes Care 26, 1790–1795 (2003). 12766111 10.2337/diacare.26.6.1790

[b4] Low C. J. . Twenty-four hour profile of the anti-hypertensive action of isradipine in essential hypertension. Blood Press 2, 59–61 (1993). 8193733 10.3109/08037059309077528

[b5] Low P. A. Testing the autonomic nervous system. Semin Neurol 23, 407–421, doi: 10.1055/s-2004-817725 (2003). 15088262

[b6] Abd El Dayem S. M., Battah A. A. & Soliman R. A. Natural progression of cardiac autonomic neuropathy in patients with type 1 diabetes: a four-year follow-up study. Anadolu Kardiyol Derg 11, 224–231, doi: 10.5152/akd.2011.061 (2011). 21466989

[b7] Vinik A. I., Maser R. E., Mitchell B. D. & Freeman R. Diabetic autonomic neuropathy. Diabetes Care 26, 1553–1579 (2003). 12716821 10.2337/diacare.26.5.1553

[b8] Pop-Busui R. What do we know and we do not know about cardiovascular autonomic neuropathy in diabetes. J Cardiovasc Transl Res 5, 463–478, doi: 10.1007/s12265-012-9367-6 (2012). 22644723 PMC3634565

[b9] Howarth F. C., Jacobson M., Shafiullah M. & Adeghate E. Long-term effects of type 2 diabetes mellitus on heart rhythm in the Goto-Kakizaki rat. Exp Physiol 93, 362–369, doi: 10.1113/expphysiol.2007.040055 (2008). 18156165

[b10] Howarth F. C., Jacobson M., Shafiullah M. & Adeghate E. Long-term effects of streptozotocin-induced diabetes on the electrocardiogram, physical activity and body temperature in rats. Exp Physiol 90, 827–835, doi: 10.1113/expphysiol.2005.031252 (2005). 16091403

[b11] Yang B. & Chon K. H. Assessment of diabetic cardiac autonomic neuropathy in type I diabetic mice. Conf Proc IEEE Eng Med Biol Soc 2011, 6560–6563, doi: 10.1109/IEMBS.2011.6091618 (2011). 22255842

[b12] Lo Giudice P. . Autonomic neuropathy in streptozotocin diabetic rats: effect of acetyl-L-carnitine. Diabetes Res Clin Pract 56, 173–180 (2002). 11947964 10.1016/s0168-8227(01)00375-8

[b13] Schmidt R. E., Nelson J. S. & Johnson E. M. Jr. Experimental diabetic autonomic neuropathy. Am J Pathol 103, 210–225 (1981). 6453533 PMC1903833

[b14] Schmidt R. E. Autonomic neuropathy in experimental models of diabetes mellitus. Handb Clin Neurol 126, 579–602, doi: 10.1016/B978-0-444-53480-4.00038-2 (2014). 25410245

[b15] Schmidt R. E. & Plurad S. B. Ultrastructural and biochemical characterization of autonomic neuropathy in rats with chronic streptozotocin diabetes. J Neuropathol Exp Neurol 45, 525–544 (1986). 3746343 10.1097/00005072-198609000-00004

[b16] O’Brien P. D. . BTBR ob/ob mice as a novel diabetic neuropathy model: Neurological characterization and gene expression analyses. Neurobiol Dis 73, 348–355, doi: 10.1016/j.nbd.2014.10.015 (2015). 25447227 PMC4416075

[b17] O’Brien P. D. . Gender-specific differences in diabetic neuropathy in BTBR ob/ob mice. J Diabetes Complications, doi: 10.1016/j.jdiacomp.2015.09.018 (2015). PMC4698064 26525588

[b18] Cheng H. T. . Increased axonal regeneration and swellings in intraepidermal nerve fibers characterize painful phenotypes of diabetic neuropathy. J Pain 14, 941–947, doi: 10.1016/j.jpain.2013.03.005 (2013). 23685187 PMC3994562

[b19] O’Brien P. D., Sakowski S. A. & Feldman E. L. Mouse models of diabetic neuropathy. ILAR J 54, 259–272, doi: 10.1093/ilar/ilt052 (2014). 24615439 PMC3962259

[b20] Sullivan K. A. . Mouse models of diabetic neuropathy. Neurobiol Dis 28, 276–285, doi: S0969-9961(07)00166-0 [pii] 10.1016/j.nbd.2007.07.022 (2007). 17804249 PMC3730836

[b21] Biessels G. J. . Phenotyping animal models of diabetic neuropathy: a consensus statement of the diabetic neuropathy study group of the EASD (Neurodiab). J Peripher Nerv Syst 19, 77–87, doi: 10.1111/jns5.12072 (2014). 24934510 PMC4303044

[b22] Johnson M. S., Ryals J. M. & Wright D. E. Early loss of peptidergic intraepidermal nerve fibers in an STZ-induced mouse model of insensate diabetic neuropathy. Pain 140, 35–47, doi: 10.1016/j.pain.2008.07.007 (2008). 18762382 PMC2602970

[b23] Jolivalt C. G. . Peripheral Neuropathy in Mouse Models of Diabetes. Curr Protoc Mouse Biol 6, 223–255, doi: 10.1002/cpmo.11 (2016). 27584552 PMC5023323

[b24] Calcutt N. A., Jorge M. C., Yaksh T. L. & Chaplan S. R. Tactile allodynia and formalin hyperalgesia in streptozotocin-diabetic rats: effects of insulin, aldose reductase inhibition and lidocaine. Pain 68, 293–299 (1996). 9121817 10.1016/s0304-3959(96)03201-0

[b25] Ingalls A. M., Dickie M. M. & Snell G. D. Obese, a new mutation in the house mouse. J Hered 41, 317–318 (1950). 14824537 10.1093/oxfordjournals.jhered.a106073

[b26] Hummel K. P., Dickie M. M. & Coleman D. L. Diabetes, a new mutation in the mouse. Science 153, 1127–1128 (1966). 5918576 10.1126/science.153.3740.1127

[b27] Sima A. A. & Robertson D. M. Peripheral neuropathy in mutant diabetic mouse [C57BL/Ks (db/db)]. Acta Neuropathol 41, 85–89 (1978). 636848 10.1007/BF00689757

[b28] Robertson D. M. & Sima A. A. Diabetic neuropathy in the mutant mouse [C57BL/ks(db/db)]: a morphometric study. Diabetes 29, 60–67 (1980). 6991317 10.2337/diab.29.1.60

[b29] Vincent A. M., Calabek B., Roberts L. & Feldman E. L. Biology of diabetic neuropathy. Handb Clin Neurol 115, 591–606, doi: 10.1016/B978-0-444-52902-2.00034-5 (2013). 23931804

[b30] Nejsum L. N. . Functional requirement of aquaporin-5 in plasma membranes of sweat glands. Proc Natl Acad Sci USA 99, 511–516, doi: 10.1073/pnas.012588099 (2002). 11773623 PMC117591

[b31] Provitera V. . Evaluation of sudomotor function in diabetes using the dynamic sweat test. Neurology 74, 50–56, doi: 10.1212/WNL.0b013e3181c7da4b (2010). 20038772

[b32] Kihara M., Opfer-Gehrking T. L. & Low P. A. Comparison of directly stimulated with axon-reflex-mediated sudomotor responses in human subjects and in patients with diabetes. Muscle Nerve 16, 655–660, doi: 10.1002/mus.880160612 (1993). 8502263

[b33] Stables C. L., Glasser R. L. & Feldman E. L. Diabetic cardiac autonomic neuropathy: insights from animal models. Auton Neurosci 177, 74–80, doi: 10.1016/j.autneu.2013.03.001 (2013). 23562143

[b34] Tessari F., Travagli R. A., Zanoni R. & Prosdocimi M. Effects of long-term diabetes and treatment with gangliosides on cardiac sympathetic innervation: a biochemical and functional study in mice. J Diabet Complications 2, 34–37 (1988). 2968355 10.1016/0891-6632(88)90026-8

[b35] http://diabetes.niddk.nih.gov/dm/pubs/neuropathies/. (Date of access: 26/11/2016).

[b36] Goncalves A. C. . Diabetic hypertensive leptin receptor-deficient db/db mice develop cardioregulatory autonomic dysfunction. Hypertension 53, 387–392, doi: 10.1161/HYPERTENSIONAHA.108.124776 (2009). 19029483

[b37] Senador D., Kanakamedala K., Irigoyen M. C., Morris M. & Elased K. M. Cardiovascular and autonomic phenotype of db/db diabetic mice. Exp Physiol 94, 648–658, doi: 10.1113/expphysiol.2008.046474 (2009). 19218356 PMC3112055

[b38] Poornima I. G., Parikh P. & Shannon R. P. Diabetic cardiomyopathy: the search for a unifying hypothesis. Circ Res 98, 596–605, doi: 10.1161/01.RES.0000207406.94146.c2 (2006). 16543510

[b39] Boudina S. & Abel E. D. Diabetic cardiomyopathy, causes and effects. Rev Endocr Metab Disord 11, 31–39, doi: 10.1007/s11154-010-9131-7 (2010). 20180026 PMC2914514

[b40] Jia G., DeMarco V. G. & Sowers J. R. Insulin resistance and hyperinsulinaemia in diabetic cardiomyopathy. Nat Rev Endocrinol 12, 144–153, doi: 10.1038/nrendo.2015.216 (2016). 26678809 PMC4753054

[b41] Felicio J. S. . Present insights on cardiomyopathy in diabetic patients. Curr Diabetes Rev (2015). 10.2174/1573399812666150914120529 PMC5101638 26364799

[b42] Ebenezer G. & Polydefkis M. Epidermal innervation in diabetes. Handb Clin Neurol 126, 261–274, doi: 10.1016/B978-0-444-53480-4.00020-5 (2014). 25410228

[b43] Ebenezer G. J., Hauer P., Gibbons C., McArthur J. C. & Polydefkis M. Assessment of epidermal nerve fibers: a new diagnostic and predictive tool for peripheral neuropathies. J Neuropathol Exp Neurol 66, 1059–1073, doi: 10.1097/nen.0b013e31815c8989 (2007). 18090915

[b44] Ebenezer G. J. . Denervation of skin in neuropathies: the sequence of axonal and Schwann cell changes in skin biopsies. Brain 130, 2703–2714, doi: 10.1093/brain/awm199 (2007). 17898011

[b45] Polydefkis M. . Safety and efficacy of ranirestat in patients with mild-to-moderate diabetic sensorimotor polyneuropathy. J Peripher Nerv Syst, doi: 10.1111/jns.12138 (2015). 26313450

[b46] Polydefkis M., Griffin J. W. & McArthur J. New insights into diabetic polyneuropathy. JAMA 290, 1371–1376, doi: 10.1001/jama.290.10.1371 (2003). 12966130

[b47] Sumner C. J., Sheth S., Griffin J. W., Cornblath D. R. & Polydefkis M. The spectrum of neuropathy in diabetes and impaired glucose tolerance. Neurology 60, 108–111 (2003). 12525727 10.1212/wnl.60.1.108

[b48] Lauria G. . European Federation of Neurological Societies/Peripheral Nerve Society Guideline on the use of skin biopsy in the diagnosis of small fiber neuropathy. Report of a joint task force of the European Federation of Neurological Societies and the Peripheral Nerve Society. Eur J Neurol 17, 903–912, e944-909, doi: 10.1111/j.1468-1331.2010.03023.x (2010). 20642627

[b49] Lauria G., McArthur J. C., Hauer P. E., Griffin J. W. & Cornblath D. R. Neuropathological alterations in diabetic truncal neuropathy: evaluation by skin biopsy. J Neurol Neurosurg Psychiatry 65, 762–766 (1998). 9810952 10.1136/jnnp.65.5.762 PMC2170354

[b50] Gordon Smith A. & Robinson Singleton J. Idiopathic neuropathy, prediabetes and the metabolic syndrome. J Neurol Sci 242, 9–14, doi: 10.1016/j.jns.2005.11.020 (2006). 16448668

[b51] Gibbons C. H., Illigens B. M., Wang N. & Freeman R. Quantification of sweat gland innervation: a clinical-pathologic correlation. Neurology 72, 1479–1486, doi: 10.1212/WNL.0b013e3181a2e8b8 (2009). 19398703 PMC2677479

[b52] Gibbons C. H., Illigens B. M., Wang N. & Freeman R. Quantification of sudomotor innervation: a comparison of three methods. Muscle Nerve 42, 112–119, doi: 10.1002/mus.21626 (2010). 20544913 PMC3048308

[b53] Loavenbruck A., Wendelschaefer-Crabbe G., Sandroni P. & Kennedy W. R. Quantification of sweat gland volume and innervation in neuropathy: Correlation with thermoregulatory sweat testing. Muscle Nerve 50, 528–534, doi: 10.1002/mus.24185 (2014). 24449525

[b54] Liu Y. . Factors influencing sweat gland innervation in diabetes. Neurology 84, 1652–1659, doi: 10.1212/WNL.0000000000001488 (2015). 25809300

[b55] Illigens B. M. & Gibbons C. H. Sweat testing to evaluate autonomic function. Clin Auton Res 19, 79–87, doi: 10.1007/s10286-008-0506-8 (2009). 18989618 PMC3046462

[b56] Dyck P. J., Davies J. L., Litchy W. J. & O’Brien P. C. Longitudinal assessment of diabetic polyneuropathy using a composite score in the Rochester Diabetic Neuropathy Study cohort. Neurology 49, 229–239 (1997). 9222195 10.1212/wnl.49.1.229

[b57] Peltier A. C. . Autonomic dysfunction in obstructive sleep apnea is associated with impaired glucose regulation. Sleep Med 8, 149–155, doi: 10.1016/j.sleep.2006.06.010 (2007). 17236808

[b58] Gibbons C. H., Illigens B. M., Centi J. & Freeman R. QDIRT: quantitative direct and indirect test of sudomotor function. Neurology 70, 2299–2304, doi: 10.1212/01.wnl.0000314646.49565.c0 (2008). 18541883 PMC3046813

[b59] Gerritsen J. . Glucose tolerance and other determinants of cardiovascular autonomic function: the Hoorn Study. Diabetologia 43, 561–570, doi: 10.1007/s001250051344 (2000). 10855530

[b60] Singh J. P. . Association of hyperglycemia with reduced heart rate variability (The Framingham Heart Study). Am J Cardiol 86, 309–312 (2000). 10922439 10.1016/s0002-9149(00)00920-6

[b61] Tsuji H. . Determinants of heart rate variability. J Am Coll Cardiol 28, 1539–1546 (1996). 8917269 10.1016/s0735-1097(96)00342-7

[b62] Liao D. . Association of vagal tone with serum insulin, glucose, and diabetes mellitus–The ARIC Study. Diabetes Res Clin Pract 30, 211–221 (1995). 8861461 10.1016/0168-8227(95)01190-0

[b63] The Atherosclerosis Risk in Communities (ARIC) Study: design and objectives. The ARIC investigators. Am J Epidemiol 129, 687–702 (1989). 2646917

[b64] Schroeder E. B. . Diabetes, glucose, insulin, and heart rate variability: the Atherosclerosis Risk in Communities (ARIC) study. Diabetes Care 28, 668–674 (2005). 15735206 10.2337/diacare.28.3.668

