# Supplementary material for: Sensory and autonomic function and structure in footpads of a diabetic mouse model
Source: Sci Rep. 2017 Jan 27;7:41401. doi: 10.1038/srep41401 (PMC5269750; doi:10.1038/srep41401)
Supplement: Supplementary Dataset 1 [file srep41401-s1.doc]

**Sensory and autonomic function and structure in footpads of a diabetic mouse model** Ying Liu, Blessan Sebastian, Ben Liu, Yiyue Zhang, John Fissel, Baohan Pan, Michael Polydefkis*, and Mohamed H Farah*

Department of Neurology, Johns Hopkins University School of Medicine

**Supplementary table 1. The reduction in autonomic fiber is greater than sensory fiber in db/db mice**

**
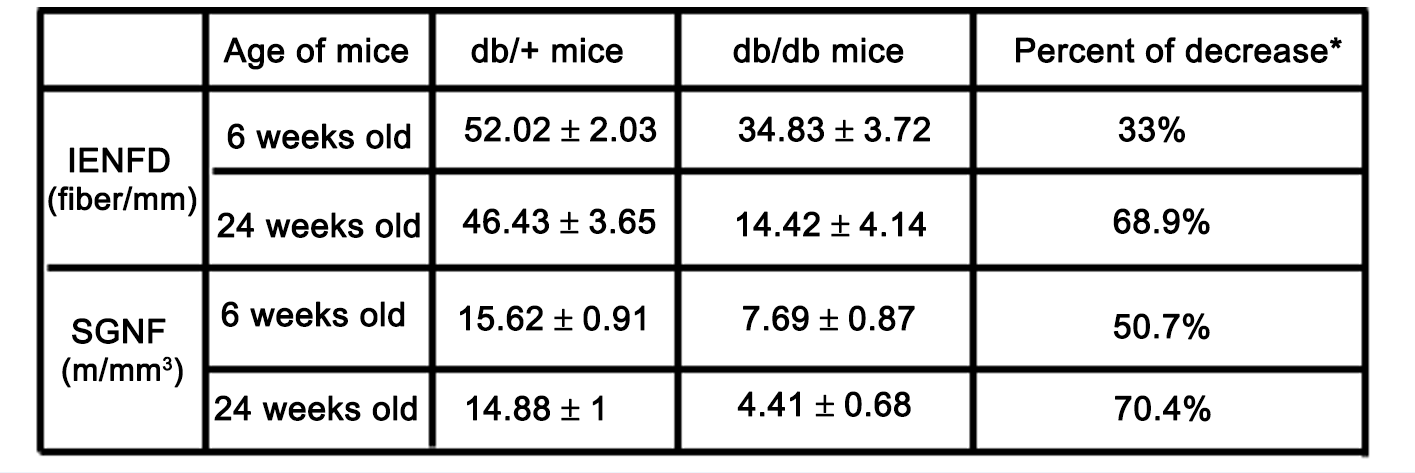
**

* Percent decrease of db/db relative to control db/+ mice.


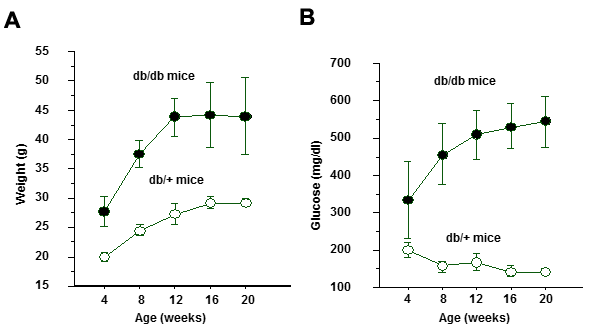
**Supplementary figures**

**Supplementary figure 1. Body weights and blood glucose levels of db/+ and db/db mice**

1. Measurements of body weights taken every 4 weeks from 4 to 20 weeks of age. Gain in body weights are much higher in db/db mice compared with db/+ mice.
2. Blood glucose levels are higher (over 300 mg/dl) in db/db mice throughout the 20 weeks.


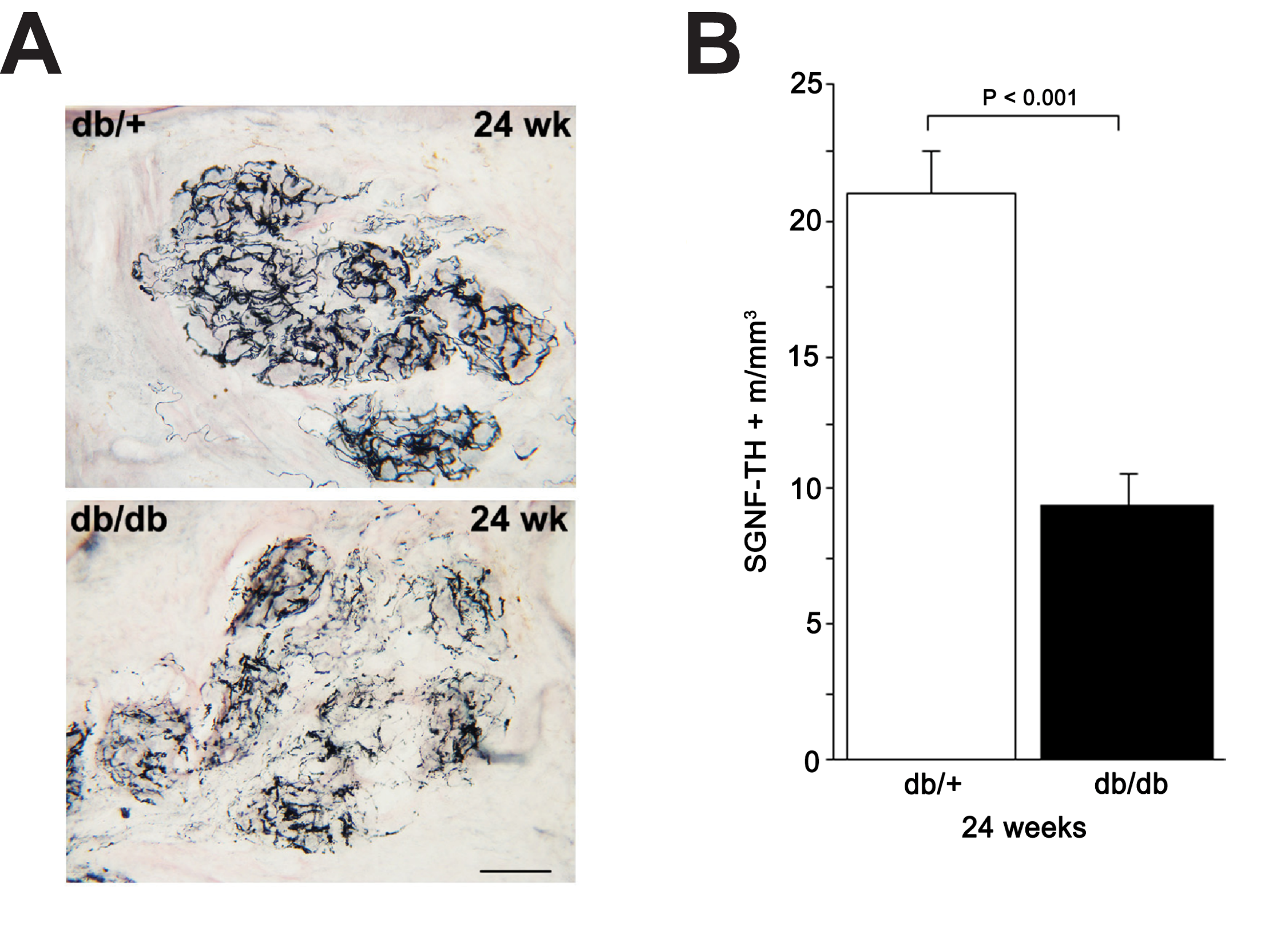


**Supplementary figure 2. Significant reduction in Tyrosine hydroxylase (TH) staining in the sweat glands of db/db mice**.

**A.** At 24 weeks of age, db/db sweat glands have drastically reduced TH staining compared to db/+ mice.

**B.** Quantification of TH staining. Db/db sweat glands have significantly less TH staining compared to db/+ sweat glands. N=5 per genotype. Scale bar + 100 μm.
